# Comparison of the Effects of Intrathecal Fentanyl and Intrathecal Morphine on Pain in Elective Total Knee Replacement Surgery

**DOI:** 10.1155/2016/3256583

**Published:** 2016-12-27

**Authors:** Refika Kılıçkaya, Yavuz Orak, Mehtap Arda Balcı, Fatih Balcı, İlker Ünal

**Affiliations:** ^1^Anesthesiology and Reanimation Clinic, Bor State Hospital, Niğde, Turkey; ^2^Anesthesiology and Reanimation Clinic, Mardin State Hospital, Mardin, Turkey; ^3^Anesthesiology and Reanimation Clinic, Niğde State Hospital, Niğde, Turkey; ^4^Medical Biostatistics Department, Çukurova University, Adana, Turkey

## Abstract

*Objective*. Total knee replacement is one of the most painful orthopedic surgical procedures. In this study, our goal was to compare the intraoperative and postoperative hemodynamic effects, the side effects, the effect on the duration of pain start, the 24-hour VAS, and the amount of additional analgesia used, of the fentanyl and morphine we added to the local anesthetic in the spinal anesthesia we administered in cases of elective knee replacement.* Materials and Methods*. After obtaining the approval of the Erciyes University Medical Faculty Clinical Drug Trials Ethics Committee, as well as the verbal and written consent of the patients, we included 50 patients in our prospective, randomized study.* Results*. In our study, the morphine group (Group M) had lower pain scores in the 2nd, 6th, 12th, and 24th hours compared to the fentanyl group (Group F). When additional analgesic requirements were compared, it was found that in the 2nd, 6th, and 24th hours fewer Group M patients needed more analgesics than did Group F patients.* Conclusion*. The fentanyl group also had lower first analgesic requirement times than did the morphine group. In terms of nausea and vomiting, there was no statistically significant difference between the two groups.

## 1. Introduction

Postoperative pain is the acute inflammatory pain that begins with the trauma of surgery and ends with the healing of the tissue. This pain has deleterious effects on organ systems and may lead to pathophysiological changes in the pulmonary/cardiovascular system [1]. The treatment of postoperative pain is crucial for homeostasis. Additionally, it has a significant impact not only on lowering the cost of treatment but also on shortening the length both of the patient's recovery time and, consequently, of her hospital stay [2, 3]. Total knee replacement is one of the most painful orthopedic surgical procedures. Patients who undergo total knee replacement are usually older and have limited cardiac and pulmonary reserves. The increased sensitivity of elderly patients to drugs makes it necessary to choose postoperative analgesia agents and methods that have minimal side effects [4]. The patients in our study were all between the ages of 60 and 90.

The purpose of this study was to compare fentanyl and morphine in terms of their intraoperative and postoperative effects, their side effects, and their effects on the onset of pain, 24-hour VAS, and the amount of additional analgesic required when they were added to the local anesthetics in the spinal anesthesia we administered to elective knee replacement patients.

## 2. Materials and Methods

After obtaining the approval of the Erciyes University Medical Faculty Clinical Drug Trials Ethics Committee, as well as the verbal and written consent of the patients, we included 50 patients in our prospective, randomized study. All were to undergo elective arthroplasty operations between July 1 and November 1, 2013; they ranged in age from 60 to 90 and were classified as ASA 1–3. Patients who had bleeding disorders; heart, liver, or renal failure; systemic infections or infections of their injection sites; psychological disorders; or drug allergies were not considered for this study. Likewise patients who did not wish to be included in the study were not considered.

All patients were visited the day before their surgery. They were given detailed information concerning the procedures about to be implemented, such as the Visual Analog Scale (VAS), sedation, nausea and vomiting, respiratory depression, and spinal anesthesia. The patients were taken to the preoperative room 30 minutes prior to the operation. A physiological infusion of 0.9% was begun intravenously via an 18-gauge intracath. The patients were given 0.02 mg/kg dormicum as a premedication. Later, in the operating room, each patient's noninvasive blood pressure (NIBP), electrocardiogram (ECG), end tidal carbon dioxide (ETCO_2_), and pulse oximetry were monitored, after which the baseline values were recorded for systolic blood pressure (SBP), diastolic blood pressure (DBP), and heart rate (HR). All patients received 2 l/min. O_2_ by nasal cannula.

Patients who met the criteria for the study were randomly divided into two groups.

The patients were seated in positions to facilitate location of intervertebral spaces and the skin was sterilized at the site where spinal anesthesia was to be administered. Following identification of either the L3-L4 or the L4-L5 interspace, a 25-gauge spinal needle was inserted midline. While Group F (*n* = 25) received 0.5% heavy bupivacaine (2.5 ml) + 25 mcg fentanyl (0.5 ml), Group M (*n* = 25) was given 0.5% heavy bupivacaine (2.5 ml) + 0.1 mg of morphine (0.5 ml) (total 3 ml for each group). As soon as the sensory block reached the appropriate level for surgery, the operation was begun. During surgery, systolic blood pressure (SBP), diastolic blood pressure (DBP), and heart rate (HR) were recorded at the 1st, 5th, 15th, 30th, and 60th minutes. At the end of each case, sensorial block was evaluated as a dermatome level using the pinprick test. Following surgery, the patients were followed up for 30 minutes in the postoperative care room before being sent back to their ward, after which their systolic blood pressure (SBP), diastolic blood pressure (DBP), heart rate (HR), nausea, vomiting, VAS values, and additional analgesic requirements (if any) were observed and recorded for the 1st, 2nd, 6th, 12th, and 24th postoperative hours. Any patient who vomited or complained of nausea was given a single 10 mg IV dose of metoclopramide. Any patient who complained of itching was given a single 50 mg IV dose of diphenhydramine HCl. A 30% reduction in systolic blood pressure was regarded as hypotension and was increased using a liquid infusion of 10 mg IV ephedrine. A heart rate of <50/min. was considered to be bradycardia and was treated with 0.5 mg IV atropine. The period of time from the moment the intrathecal injection was made postoperatively until the first analgesic became necessary was recorded as the postoperative first-analgesic requirement time and was likewise recorded.

Desaturation was defined as the falling of the SpO_2_ below 96% and was treated by administering 2 l/min. of O_2_ by mask.

Patients who complained of pain (VAS > 3) and needed analgesics were treated intramuscularly every six to eight hours with Diclofenac Na.

Postoperative pain was assessed using the Visual Analog Scale (VAS). The Visual Analog Scale is one of the methods commonly used in the evaluation of pain intensity. The VAS is a verbal scale numbered from 0 to 10, with 0 being “no pain” and 10 being “the worst pain possible or imaginable.” Accordingly, the patient is requested to verbally express his degree of pain using this scale.

### 2.1. Presentation of Statistical Analysis and Data

The* SPSS 18.0* software package, the nonparametric Mann–Whitney *U* Test, the chi-squared test, and the independent sample *t*-test were used in the statistical analysis of the data; and *p* > 0.05 was considered statistically significant.

## 3. Results

A total of 50 patients were included in the study. All of them completed the study. Their demographic data (age, height, weight, and gender) are shown in Table 1. There was no statistically significant difference (*p* < 0.05) between the two groups.

Although there was a decrease in the SBP in both groups from the 1st to the 60th intraoperative minutes, there was no statistically significant difference between the groups in terms of intraoperative systolic and diastolic blood pressure.  Table 2 shows that the change over time for the groups was similar (*p* > 0.05).

Although there was a decrease in the intraoperative pulse pressure in both groups from the 1st to the 60th operative minutes, there was no statistically significant difference between the groups.  Table 3 shows that the variations of the groups over time were similar (*p* > 0.05).

Table 4 shows that although there was a decrease in the systolic and diastolic blood pressure in both groups from the 1st to the 24th postoperative hour, there was no statistically significant difference between the groups (*p* > 0.05).

Table 5 illustrates that no statistically significant difference was observed between the groups in terms of postoperative heart rate values (*p* > 0.05).

In terms of End-of-Case Sensory Block Level measurements, a statistically significant difference between the two groups was not detected, as is shown in Table 6.

Table 7 shows that there was no statistically significant difference in first-analgesic requirement time measurements between the two groups.

As can be seen in Table 8, when postoperative VAS pain scores are compared, Group M's scores for the 2nd, 6th, 12th, and 24th hours are, statistically speaking, significantly lower than those of Group F (*p* < 0.05).

When the additional analgesic requirements of the two groups were compared, it was found that in the 2nd, 6th, and 24th hours, statistically speaking, Group M's needs were significantly lower than those of Group F.  Table 9 reflects this.

Table 10 shows that in our study there was no statistically significant difference between Groups M and F in terms of nausea and vomiting (*p* > 0.05).

## 4. Discussion

Multimodal analgesia protocol may increase analgesic activity. Severe pain can be treated with intravenous opioids and NSAIDs used as patient-controlled analgesia (PCA), epidural local anesthetics and/or opioids, techniques called peripheral nerve blocks, or different combinations of drugs [5, 6]. Of all knee replacement surgery patients, 60% describe the after-surgery pain they feel as severe, while 30% call it moderate [7]. In order to reduce analgesia and avoid side effects in these patients, use of regional anesthesia in conjunction with multimodal techniques is more effective pain control [8]. It has been reported that implementation of intrathecal morphine is effective in postoperative pain control. The analgesic effects and intrathecal side effects of morphine in the 0.0–0.3 mg dose range were studied. The result of that study was that the authors found that 0.2 mg and 0.3 mg doses of intrathecal morphine offered more effective pain relief than did 0.0 mg and 0.1 mg doses of the same in hip and knee arthroplasty. Itching, nausea, and vomiting were determined to be dependent on 0.1 mg and 0.3 mg intrathecal use and dose-dependent as discussed by Rathmell et al. [9]. On the other hand it is unacceptable to use even small doses of morphine because of these side effects as discussed by Gürkan et al. [10]. The usage of 0.5 mg intrathecal morphine was as safe as and more effective than 0.2 mg injections as discussed by Gupta [11]. Also the analgesic effect of the additional opioids provides a long postoperative period without pain. As shown in several previous studies, the patients with morphine had a long duration of analgesia [12]. This adjuvant analgesic technique is expected to decrease postoperative pain intensity and opioid requirements and to speed up recovery. Intrathecal morphine, which is less hydrophobic than other opioids, has a longer residence time in the cerebrospinal fluid and provides excellent postoperative analgesia [13]. Adjuvants are often added to local anesthetics to increase the quality of anesthesia in patients undergoing spinal anesthesia, prolong the duration of the anesthesia, and reduce the side effects of (lower dose) anesthetics. The most commonly used adjuvant drugs are opioids. Opioids, when used in combination with local anesthetics, are known to produce more effective and longer-term anesthesia [14, 15]. Time to first-analgesic requirement was also significantly longer in Group C (hyperbaric bupivacaine, fentanyl, and MgSO_4_). Total morphine consumption was significantly less in Group C. The severity of pain was significantly less in C group as discussed by Attari et al. [16]. In addition, the duration of sensory analgesia is significantly prolonged with addition of morphine ensuring neonatal well-being [17].

In another study, the qualities of postoperative analgesia between intrathecal fentanyl 25 mcg and intrathecal morphine 0.1 mg in patients undergoing cesarean section were compared. The postoperative analgesia of intrathecal fentanyl was inferior to that of intrathecal morphine discussed by Salmah and Choy [18]. Similarly, the current study found that the postoperative VAS pain scores of Group M (the morphine group) were statistically significantly lower in the 2nd, 6th, 12th, and 24th hours in comparison with those of Group F (the fentanyl group) (*p* < 0.05). Meanwhile, when the two groups were compared in terms of additional analgesics required, it was found that fewer patients in Group M, to a statistically significant degree, needed additional analgesics in the 2nd, 6th, and 24th hours compared to those of Group F. There was also a statistically significant difference in first-analgesic requirement times between the two groups: Group F had lower times than Group M. We also found no significant difference, statistically, between the two groups in terms of nausea and vomiting. It was discussed by Agrawal et al. [17] that there is reduction in the incidence of nausea, vomiting, and shivering by the addition of fentanyl to bupivacaine. It has been discussed by Saracoglu et al. [19] in the study that intrathecal 15 mg isobaric bupivacaine with 200 *µ*g morphine provides longer duration of analgesia and similar haemodynamic effects and ephedrine requirement and side effects when compared to heavy bupivacaine-morphine or bupivacaine-fentanyl combinations during cesarean section.

The additional analgesic requirement period was significantly longer in Group Morphine than in Group Fentanyl (*p* < 0.001). Intraoperative and postoperative complications were significantly higher in Group Fentanyl than in Group Morphine (*p* < 0.05). Intended, delivered, and total analgesic amount values were significantly higher in Group Fentanyl than in Group Morphine (*p* < 0.001) [20].

In our study it was found that the morphine group had lower pain scores than did the fentanyl group in the 2nd, 6th, 12th, and 24th hours. And in the 2nd, 6th, and 24th hours the morphine group was also found to have fewer patients who required additional analgesics than did the fentanyl group.

## 5. Conclusion

In conclusion, the fentanyl group had lower first-analgesic requirement times than compared to the morphine group. And there was no statistically significant difference between the two groups in terms of nausea and vomiting.

## Figures and Tables

**Table 1:** Patients' demographic data (mean ± SD).

	Group M	Group F	Value of *p*
Age (years)	70.96 ± 6.94	68.56 ± 5.50	*p* = 0.275
Height (cm)	163.24 ± 7.65	164.32 ± 7.21	*p* = 0.076
Weight (kg)	73.24 ± 17.70	76.28 ± 5.50	*p* = 0.106
Sex (M/F)	15/10	19/6	*p* = 0.225

**Table 2:** Intraoperative systolic (S) and diastolic (D) blood pressure values (mmHg) (mean ± SD).

	Group M	Group F	Value of *p*
Beginning S	163.4 ± 15.5	167.2 ± 12.8	*p* = 0.327
Beginning D	86.4 ± 14.3	91.4 ± 10.6	*p* = 0.260
5th min. S	146.6 ± 18.7	154.8 ± 12.1	*p* = 0.089
5th min. D	81.1 ± 12.0	84.1 ± 10.2	*p* = 0.251
15th min. S	130.2 ± 15.9	140.6 ± 12.2	*p* = 0.013
15th min. D	74.2 ± 12.9	74.4 ± 8.2	*p* = 0.846
30th min. S	125.9 ± 11.9	133.4 ± 10.8	*p* = 0.031
30th min. D	70.0 ± 9.9	74.2 ± 7.7	*p* = 0.127
60th min. S	124.3 ± 12.3	128.7 ± 10.9	*p* = 0.183
60th min. D	70.4 ± 8.1	73.4 ± 7.9	*p* = 0.193

**Table 3:** Intraoperative pulse pressure values (mmHg) (mean ± SD).

	Group M	Group F	Value of *p*
Beginning	83.5 ± 14.3	86.9 ± 9.8	*p* = 0.299
5th min.	78.9 ± 11.0	83.0 ± 10.8	*p* = 0.248
15th min.	76.4 ± 10.3	78.0 ± 7.5	*p* = 0.593
30th min.	74.8 ± 9.5	75.5 ± 8.2	*p* = 0.846
60th min.	78.3 ± 10.9	74.1 ± 8.2	*p* = 0.150

**Table 4:** Postoperative systolic blood pressure (SBP) and diastolic blood pressure (DBP) values (mmHg) (mean ± SD).

	Group M	Group F	Value of *p*
1st hr. SBP	123.6 ± 14.5	121.4 ± 20.7	*p* = 0.783
1st hr. DBP	72.6 ± 7.7	71.8 ± 12.9	*p* = 0.952
2nd hr. SBP	126.6 ± 12.9	120.0 ± 19.7	*p* = 0.124
2nd hr. DBP	73.0 ± 6.8	73.2 ± 10.5	*p* = 0.867
6th hr. SBP	125.7 ± 10.4	125.0 + 14.8	*p* = 0.847
6th hr. DBP	74.1 ± 8.1	73.2 ± 9.8	*p* = 0.761
12th hr. SBP	127.0 ± 0.0	124.8 ± 12.9	*p* = 0.734
12th hr. DBP	75.6 ± 7.0	74.1 ± 9.4	*p* = 0.694
24th hr. SBP	126.6 ± 6.5	132.4 ± 7.9	*p* = 0.005
24th hr. DBP	73.6 ± 9.6	77.4 ± 7.1	*p* = 0.125

**Table 5:** Postoperative heart rate values (beats/min) (mean ± SD).

	Group M	Group F	Value of *p*
1st hr.	77.8 ± 5.9	78.4 ± 5.7	*p* = 0.840
2nd hr.	77.0 ± 5.8	77.2 ± 6.8	*p* = 0.590
6th hr.	76.7 ± 6.0	77.7 ± 6.8	*p* = 0.993
12th hr.	77.5 ± 12.5	75.9 ± 6.4	*p* = 0.757
24th hr.	76.8 ± 8.5	76.8 ± 6.8	*p* = 0.792

**Table 6:** End-of-Case Sensory Block Level (T).

	Group M (T8-T9)	Group F (T8-T9)	Value of *p*
End-of-Case Sensory Block Level	8.7 ± 0.9	8.8 ± 0.9	*p* = 0.783

**Table 7:** First analgesic requirement time.

	Group M	Group F	Value of *p*
First analgesic requirement time	5.9 ± 1.3	2.6 ± 0.6	*p* < 0.001

**Table 8:** Postoperative Visual Analog Scale (VAS) pain score values (Mean ± SD).

	Group M	Group F	Value of *p*
1st hr.	0.4 ± 0.2	0.8 ± 0.4	*p* = 0.977
2nd hr.	0.2 ± 0.7^*∗*^	1.6 ± 2.1^*∗*^	*p* = 0.006
6th hr.	4.2 ± 1.6^*∗*^	6.7 ± 1.6^*∗*^	*p* = 0.001
12th hr.	3.2 ± 1.7^*∗*^	5.6 ± 1.5^*∗*^	*p* = 0.001
24th hr.	1.3 ± 1.3^*∗*^	4.1 ± 0.5^*∗*^	*p* = 0.001

0 = no pain; 10 = worst possible pain.

^*∗*^ *p* < 0.05.

**Table 9:** Additional analgesic needs of the groups (*n* (%)).

	Group M	Group F	Value of *p*
1st hr.	0 (0%)	0 (0%)	
2nd hr.	0 (0%)^*∗*^	7 (28%)^*∗*^	*p* = 0.004
6th hr.	14 (56%)^*∗*^	24 (96%)^*∗*^	*p* = 0.001
12th hr.	19 (76%)	22 (88%)	*p* = 0.269
24th hr.	6 (24%)^*∗*^	23 (92%)^*∗*^	*p* < 0.001

^*∗*^ *p* < 0.05. Group M, as compared to Group F.

**Table 10:** Nausea/vomiting.

	Group M	Group F	Value of *p*
1st hr.	1 (4%)	3 (12%)	*p* = 0.297
2nd hr.	2 (8%)	3 (12%)	*p* = 0.637
6th hr.	1 (4%)	1 (4%)	*p* = 0.000
12th hr.	1 (4%)	1 (4%)	*p* = 0.312
24th hr.	0 (0%)	0 (0%)	
